# Forming Control and Wear Behavior of M2 High-Speed Steel Produced by Direct Energy Deposition on Curved Surface

**DOI:** 10.3390/ma17246119

**Published:** 2024-12-14

**Authors:** Lan Jiang, Xiaofang Pan, Zhongkai Li, Bo Yuan, Wenxin Liu, Danya Li, Ge Shen, Jun Liu

**Affiliations:** School of Materials Science and Engineering, Central South University, Changsha 410083, China

**Keywords:** direct energy deposition, curved surface deposition, roller die cutter, M2 high-speed steel, wear resistance

## Abstract

Direct energy deposition (DED) technology shows promising applications in the production of roller die cutters. The optimization of process parameters, scanning strategies, and analyses of compressive properties and wear behavior are required prior to application. Therefore, this work investigated the influence of scanning strategy and overlap ratio on the microstructure, microhardness, compressive properties, and wear resistance of M2 high-speed steel (HSS) with DED on a 316 L cylindrical surface. The results reveal that along the deposition direction of the sample, the grain size gradually decreases, with hardness increasing from 187 HV in the matrix to 708 HV. As the overlap ratio increases, the grain size initially rises and then decreases, while hardness first declines and subsequently increases. The cross-scanning strategy effectively enhances the compressive strength by reducing porosity defects. Furthermore, the compressive strength of the samples initially increases with the overlap ratio before experiencing a slight decrease. The M-3 sample with a 50% overlap ratio exhibits the best compressive strength (3904 MPa). The wear rate decreases and then increases with the rising overlap ratio. Therefore, the M-3 sample, prepared using cross-scanning strategies with an overlap ratio of 50%, demonstrates a uniform and dense microstructure, resulting in superior wear resistance, and the wear rate is as low as 8 × 10^−6^ mm^3^·N^−1^·m^−1^. The current experimental results provide valuable references for the DED of die-cut knives.

## 1. Introduction

Die-cutting tools occupy a pivotal position in the field of molds, widely used in electronics [1], packaging [2], printing [3], and other industries. These die-cutting tools have become an indispensable part of modern industrial production. With the rapid advancements in modern technology, the die-cutting industry is gradually transitioning towards high efficiency, high precision, high quality, and intelligent development [4]. Die-cutting tools can be mainly classified into two main types: roller die cutters and flat die cutters. The roller die cutter has largely replaced the flat die cutter because of its flexibility, high efficiency, and high precision [5]. However, the roller die-cutting machine operates by applying pressure between two rollers, resulting in significant pressure on the cutting surface of the die. During operation, the die-cutting knives may be subjected to impacts and vibrations, leading to wear, chipping, or even breakage over time, which in turn affects the precision and quality of die-cutting products [6]. Therefore, selecting high-strength, high-hardness, wear-resistant materials with sufficient toughness for the fabrication of die-cutting knives is crucial to ensure the longevity of the dies. M2 high-speed steel (HSS) is a kind of cold work die steel which has a high content of alloying elements such as W, Mo, Cr, and V. These elements form numerous hard carbides in the microstructure, granting M2 steel excellent hardness, toughness, and wear resistance, making it widely used in the production of various dies [7].

Currently, die-cutting tools are primarily fabricated by subtractive methods such as cutting and engraving, which require multiple processes including heat treatment, making the manufacturing process complex, time-consuming, and costly [8]. Consequently, in order to promote the efficient and high-quality development of the die-cutting industry, it is imperative to explore new methods for die-cutting tools manufacturing. The potential methods for preparing thin metal layers mainly include physical vapor deposition, chemical vapor deposition, thermal spraying, laser additive manufacturing, and so on. However, the low efficiency of physical vapor deposition, the complex process of chemical vapor deposition, and the unstable quality of thermal spraying limit its application in this field. Metal laser additive manufacturing technology primarily based on selective laser melting (SLM) [9] and direct energy deposition (DED) has transformed the traditional processing mode and realized the integrated forming of metals. DED melts the powder on the surface of the substrate instantaneously by a fast-moving high-energy laser beam, and then the powder rapidly cools and solidifies. The deposition layer repeatedly undergoes a heating–cooling cycle to build a three-dimensional component from the bottom to the top [10]. This technique has been widely applied in fabricating complex-shaped parts [11], repair [12], welding [13], and surface strengthening [14]. Compared to traditional forming methods, DED technology leads to grain refinement due to its non-equilibrium solidification and undercooling, significantly improving the hardness and wear resistance of the material [15]. Thus, DED holds great potential for application in the production and repair of die-cutting knives. By employing DED to deposit appropriate materials on the surface of the rollers and then sharpening, material waste can be minimized, and the requirements for heat treatment can be eliminated, thereby reducing energy consumption while meeting the high hardness, wear resistance, and minimal demand requirements of the dies, ensuring cutting quality. It is worth noting that DED can achieve good metallurgical bonding with dissimilar metals [16], which means that the performance requirements of the round die materials are reduced. At the same time, the hardness of the prepared die-cutting knives is significantly higher than that of the cutter body, providing a longer die-cutting life. Although the DED has significant application potential in the preparation of circular die-cutting knives and there is considerable research on DED of M2 HSS [17,18,19], there are currently limited studies on the practical application of DED in die-cutting tools.

At present, the research on DED mainly focuses on flat and inclined surfaces, with limited attention being paid to cylindrical surfaces. Additionally, there is evidence that process parameters and scanning strategies have significant effects on microstructure, defect control, and performance [20,21,22]. Several researchers have explored the effects of laser power, scanning rate, and powder feeding rate on deposited samples [23,24,25,26]. Kim et al. [27] used DED to prepare WC-12Co cemented carbide. It was found that reducing the hatch spacing can reduce the pores and improve the compressive strength of the sample. In another study, Bu et al. [28] demonstrated that samples with higher overlap ratios exhibited finer microstructures and better wear resistance when depositing chromium-rich stainless steel using high-speed DED. J H et al. [29] found that too high an overlap rate during the directed energy deposition of Inconel 718 caused excessive molten pool, while too low an overlap rate will easily lead to fractures and pores in the sample. Furthermore, the microstructure of the samples prepared by different scanning strategies exhibits obvious differences, which in turn affects its tensile properties. Pang et al. [30] investigated the effects of the building strategies on the microstructure and mechanical properties of the samples in DED. The grain size of the sample scanned by long unidirectional grating is smaller, and the tensile strength and yield strength are superior. Das et al. [31] measured the wear resistance of 15-5PH stainless steel deposited by three different scanning strategies, and the results indicated that scanning strategies had a significant impact on wear resistance, with the island-alternating strategy showing the best wear resistance. During the operation of cylindrical die-cutting machines, the contact area between the cutting edge and the pressure roller is very small, resulting in high pressure on the cutting edge. At the same time, sliding friction occurs between the cutting edge and the product, which can easily lead to abrasion. Therefore, the cutting edge of die-cutting tools must have high compressive strength and wear resistance. To ensure the durability and reliability of cylindrical dies, exploring laser strategies and overlap ratios during DED is inevitable.

In this study, M2 HSS was directly deposited on the surface of a 316 L cylinder using different scanning strategies and overlap ratios. The microstructure, phase composition, element distribution, and microhardness of the deposited samples were characterized and analyzed. In addition, compression tests and wear resistance tests were conducted to comprehensively analyze the effects of scanning strategies and overlap ratios on the defect control, compressive performance, and wear resistance of M2 HSS deposited on cylindrical surfaces. This study aims to support the application of DED in the fabrication of cylindrical die-cutting tools.

## 2. Materials and Methods

### 2.1. Characterization of M2 Powder

In this study, M2 HSS spherical powder prepared by gas atomization was produced by Hunan Hualiu New Material Co., Ltd. (Changsha, China). Table 1 shows the main composition of the powder. The M2 powder with a particle size of 75–150 μm was screened by a standard sieve. Then, the powder characteristics were analyzed after the screened powder was vacuum dried.

In order to test the quality of M2 powder, the surface and cross-section morphology of M2 powder were observed using an optical microscope (OM). The microstructure and element distribution of the powder’s surface and cross-section were investigated using a Tescan Mira 4 scanning electron microscope (SEM, Tescan Orsay Holding, Brno, Czech Republic) equipped with an energy dispersive spectrometer (EDS). The particle size distribution of the powder was evaluated using a Mastersizer 3000 laser diffraction particle size analyzer (Malvern Panalytical, Malvern, UK). The chemical composition and phase of the powder were analyzed using a SmartLab SE physical X-ray diffractometer (Rigaku Corporation, Tokyo, Japan) with Cu Kα radiation.

### 2.2. Direct Energy Deposition

**Figure 1:**
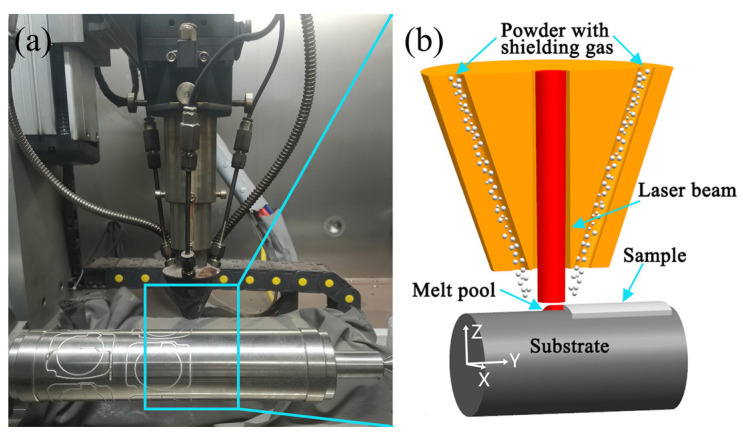
DED experiment: (**a**) laser generator and (**b**) schematic diagram of deposition process.

The experiment employed a self-assembled coaxial powder feeding DED system, which consists of a fiber laser generator, a five-axis CNC system, a coaxial powder feeding system, a gas purification system, and a water-cooling system. The laser spot diameter produced by the laser generator (Figure 1a) is 2 mm, with a maximum laser power of 2 kW. The DED process is illustrated in Figure 1b, where argon gas transports the powder to the substrate surface, and the high-energy laser beam melts the powder and the substrate surface, forming a good metallurgical bond between the sample and the substrate. To prevent oxidation of the powder during the deposition process, argon gas with a purity of 99.9% was used as the protective and powder carrier gas, and the experiments were conducted in an argon-filled glove compartment to ensure that the water and oxygen content inside the compartment remained below 500 ppm.

A 300 mm diameter 316 L stainless steel rod was selected for DED, with a hardness of approximately 190 HV. The chemical composition of the steel rod is shown in Table 2. The steel rod was cleaned with anhydrous ethanol, and after complete drying, M2 HSS was deposited on its surface. In order to prepare a dense and uniform sample, a total of 27 single tracks were deposited with a full factorial design of experiment designing laser power, scanning speed, and powder feeding rate at three levels to measure their geometric characteristics. The combination of process parameters with the minimum dilution rate, the highest deposition efficiency, and the best accuracy was selected: 500 W laser power, 400 mm/min scanning speed, and 0.4 r/min powder feeding rate.

**Figure 2:**
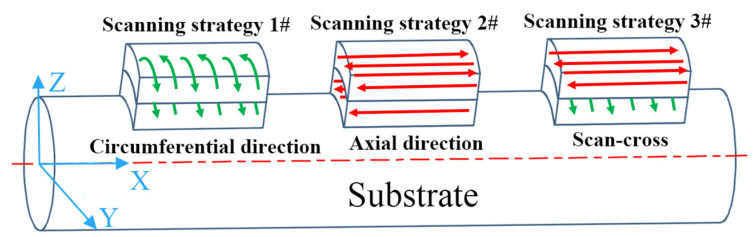
Laser scanning strategies diagram.

According to the existing research, five groups of deposition experiments with different scanning strategies and overlap ratios were designed to further enhance the performance of M2 HSS, with two samples of dimensions 15 mm (W) × 10 mm (D) × 8 mm (H) prepared for each group. It has been reported that samples with bidirectional scanning exhibit strong fiber texture and lower residual stress, thus demonstrating superior mechanical properties; therefore, a bidirectional scanning method was employed for each layer [32,33,34]. The schematic diagram of different scanning strategies is shown in Figure 2. Scanning strategy 1# means that the scanning direction is along the circumferential direction of the cylinder. And scanning strategy 2# means that the scanning direction is parallel to the axial direction of the cylinder. Scanning strategy 3# refers to the 90° cross-scanning of the adjacent layer where the N layer is scanned along the axial direction of the substrate and the N + 1 layer is scanned parallel to the axial direction of the substrate, with each layer raised by 0.2 mm in the *Z*-axis. Table 3 presents the combination of processing parameters of deposition samples. The first three groups of samples have different scanning strategies, while the last three groups have different overlap ratios. In DED, the relationship between scanning spacing and overlap ratio can be expressed as follows:

S = w × (1 − η),(1)

where S is the scanning spacing, w is the width of the single-pass deposition layer, and η is the overlap ratio.

### 2.3. Detection of M2 Deposited Samples

**Figure 3:**
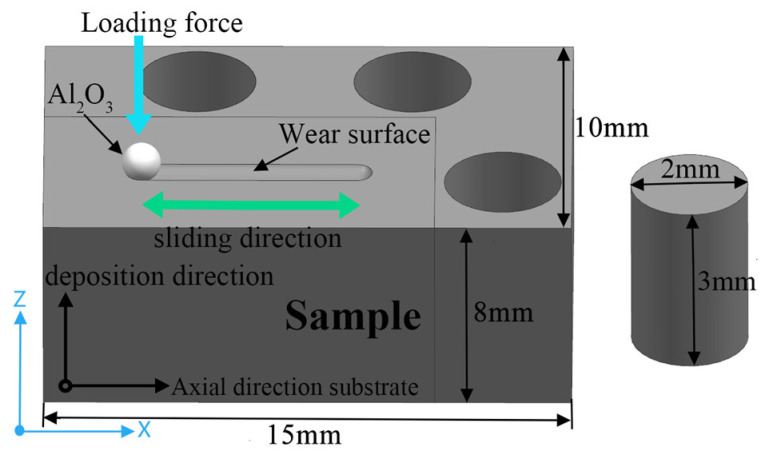
The segmentation diagram of the deposited sample.

Considering that the cutter body prepared by DED can be used for die-cutting products after simple machining, the hardness test and wear resistance test are selected at the top of the deposited sample. The M2 block was first cut along the deposition direction using wire cutting technology to obtain wear-resistant and compressed test samples according to Figure 3, which were polished with sandpaper, followed by polishing with diamond abrasives. After etching with aqua regia for 2 to 4 s, the samples were cleaned and dried. The microstructure and element distribution of the samples were observed using an OM and a SEM. X-ray diffraction (XRD) was used to perform phase identification on the samples, scanning at a rate of 5°/min. The hardness of the samples was measured on the surface free of noticeable scratches using the HMAS-D1000Z Vickers (Hunan Qinquan Inspection and Testing Instrument Co., Ltd., Changsha, China) hardness tester, with 13 indentation tests conducted for each sample to minimize experimental error. In addition, the hardness distribution of the representative sample M-1 in the cross-section was measured. The hardness test was performed every 0.6 mm from the top of the deposition layer to the substrate, testing six points horizontally at each position.

Quasi-static unidirectional mechanical compression tests were conducted using a universal mechanical testing machine (Jinan Hengxu Testing Machine Technology Co., Ltd., Jinan, China). In order to reduce experimental errors, three groups were performed to evaluate the compression performance at room temperature, and the median results were selected for comparative analysis. Since the compression direction for the cutting edge is the radial direction of the cylinder, a cylinder with a diameter of 2 mm and a height of 3 mm were cut along the radial direction as a compression sample, and the compression rate was set at 0.2 mm/min. The linear reciprocating wear resistance test of the samples with surfaces free of noticeable scratches was tested using a UMT-3 multifunctional friction (Computers and Electronics in Tribology Research, Campbell, CA, USA) and wear tester in the atmosphere at room temperature. Table 4 presents the specific wear testing parameters. The wear surface was scanned using a MicroXAM-3D (Bruker Corporation, Billerica, MA, USA) white light interference three-dimensional surface profiler, and the wear rate (W_v_, mm^3^·N^−1^·m^−1^) was calculated by the following formula.

W_v_ = V/(P × S), (2)

where V represents the wear volume (mm^3^), P is the test load (N), and S is the total sliding distance (m).

## 3. Results and Discussion

### 3.1. Powder Characteristic

**Figure 4:**
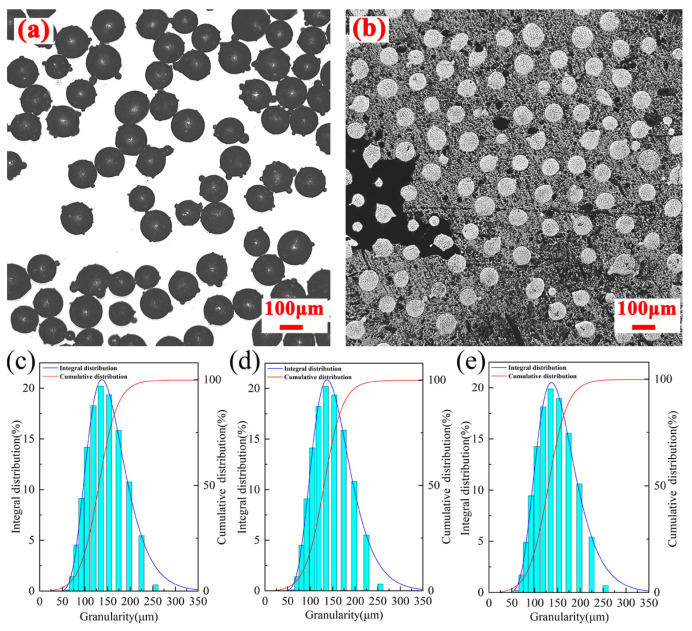
Morphology and particle size distribution of M2 powder: (**a**) surface, (**b**) cross-section, and (**c**–**e**) three wet particle size distribution test results.

Gas atomization is a commonly used method for preparing powders in DED, where metal melts are broken by high-speed gas jets and then solidified. Compared to water-atomized powders, gas-atomized powders exhibit better sphericity and fluidity, with fewer powder splashing events during DED [35]. High-quality powder is essential to ensure the performance of components produced via DED. Studies have shown that powders with higher sphericity possess better fluidity, leading to higher density in both the powder and the corresponding deposited samples [36]. The optical microscopy images of the surface and cross-sections of M2 powder are shown in Figure 4a,b. As shown in Figure 4a, the diameter of the powder particles ranges from 111 to 154 nm. The images reveal that, apart from a small portion of satellite powder, most M2 powder particles exhibit good sphericity, and only a small proportion of the powder shows internal defects, overall indicating good powder quality.

To ensure the accuracy of the powder particle size distribution, wet particle size distribution measurements were conducted three times, and the results were plotted in Figure 4c–e. The powder particle size distribution is relatively concentrated, and generally follows a normal distribution. It is well known that the poor fluidity and filling performance of the powder may lead to internal defects such as pores and voids in deposited samples. According to Zhang et al. [37], particle size distribution plays a significant role in improving the fluidity of the powder, with both a concentrated distribution and larger average particle size enhancing flowability. Moreover, a large number of studies indicate that the microstructure and micro-segregation of gas-atomized powders are closely related to particle size, and excessively large or small sizes are unfavorable for achieving a uniform and dense microstructure [38,39]. Table 5 presents the values of d10, d50, and d90 distribution diameters, with the three measurements showing basically consistent results. The average particle size is about 130 μm, which is slightly larger than the nominal particle size reported by the manufacturer.

**Figure 5:**
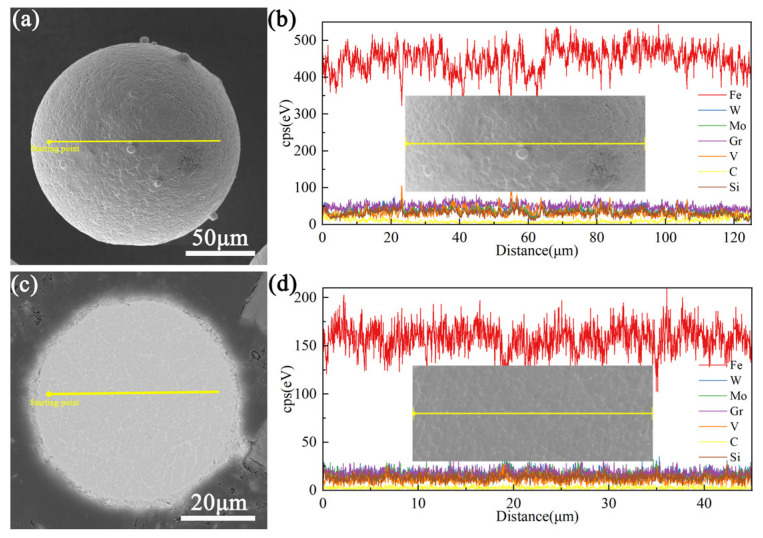
Results of elemental line distribution: (**a**,**b**) surface and (**c**,**d**) cross-section.

**Figure 6:**
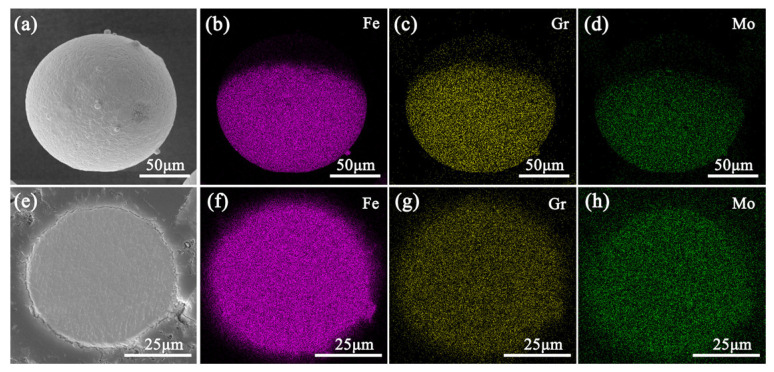
Results of elemental area distribution: (**a**–**d**) surface and (**e**–**h**) cross-section.

Due to the complex chemical composition of M2 HSS, the excessive segregation of elements has an adverse effect on the properties of the deposited components. Therefore, it is necessary to analyze the elemental distribution of individual powder particles using an EDS. Figure 5 shows the linear distribution of elements on the surface and cross-section of M2 powder particles. Aside from slight fluctuations in Fe content, the distribution of other elements is relatively uniform. The grain size in the cross-section is approximately 4 μm. In order to understand the element distribution of the powder more comprehensively, EDS elemental mapping was performed on the surface and cross-sections of the powder, as shown in Figure 6. This further demonstrates that there is no obvious element segregation on the surface and cross-section of the powder. The microscopic observation of individual powder particles reveals a fine honeycomb and petal cell mixed structure on the surface, with a small amount of shrinkage micropores, and a clear honeycomb cell crystal structure inside. At the same time, a continuous network of white phases is distributed along the crystal boundary. The morphology of the powder is related to the evolution of the microstructure, which depends on the thermal history during solidification [40]. The surface temperature gradient of the spherical powder particles is the largest, with isotropic heat transfer conditions. As a result, nucleation occurs first on the surface, and the grains grow toward the center. Due to the high degree of supercooling and the release of the latent heat of crystallization, nuclei form inside the particles, and the nuclei grow at the same rate in all directions. Different heat and mass transfer conditions have an important influence on the morphology of the alloy solidified structure. In the gas atomization process, the rapid non-equilibrium solidification process causes the cell structure to develop into equiaxed grains instead of dendrites. Therefore, the microstructure of the gas-atomized M2 HSS powder is dominated by equiaxed grains.

### 3.2. Phase Composition

The XRD patterns of the M-1 sample and M2 powder are shown. The powder samples mainly consist of α-Fe, a small amount of γ-Fe, M_2_C, and MC-type primary eutectic carbides. M_2_C is a metastable carbide containing W and Mo, while MC is mainly composed of high-hardness WC, MoC, VC, etc., which can improve wear resistance. In the process of non-equilibrium rapid solidification, M2 HSS generally forms ferrite first, which then transforms into austenite. Upon further cooling, austenite undergoes martensitic transformation. Therefore, the phase composition of the powder prepared by gas atomization is closely related to particle size. HSS powder with larger particle size contains more austenite due to its slower cooling rate, while the powder with a small particle size contains more ferrite. In addition, the solubility of carbon in ferrite is relatively low, and the carbon element will be repelled to the equiaxed grain boundary to form network carbides with alloy elements during the solidification of HSS.

**Figure 7:**
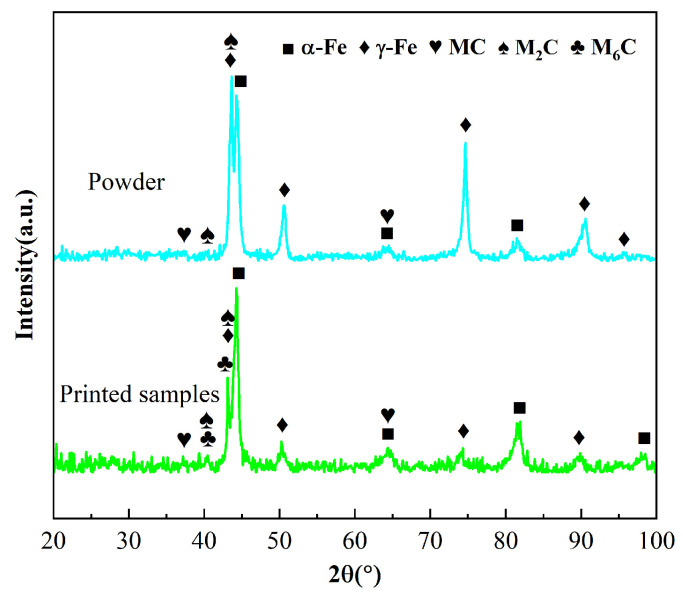
XRD spectra of powder and deposited samples.

As shown in Figure 7, the deposited samples contain martensite, retained austenite, M_6_C, M_2_C, and MC-type carbides. M_6_C is a ternary eutectic carbide rich in Fe, W, Mo, and Cr. Different from simple cubic structure of MC, M_6_C belongs to the class of complex cubic structured carbides. It is either primary carbide precipitated from austenite during the solidification process or secondary carbide formed from the decomposition of M_2_C. During the rapid melting and solidification of the powder in DED, the temperature easily reaches the martensite transformation point, promoting a martensitic transformation of austenite, thus making high-hardness martensite the main matrix phase of the deposited samples. A small amount of austenite is retained in the sample, which is due to the fact that the cooling rate is too fast, inhibiting atomic diffusion, preventing the austenite from completely transforming into martensite [41]. The carbon and alloying elements in the high-speed steel melt cannot be dissolved completely in time, resulting in the segregation of elements to form M_6_C, M_2_C, and MC carbides. Additionally, the short cooling time causes the metastable M_2_C carbides to not fully decompose into the stable MC and M_6_C carbides. The precipitated eutectic carbides are hard and brittle phases, and their morphology, size, and distribution significantly affect the mechanical properties of the deposited M2. The higher degree of carbide spheres, smaller size, and more uniform distribution are more favorable for enhancing the strength of M2.

### 3.3. Microstructure and Microhardness

**Figure 8:**
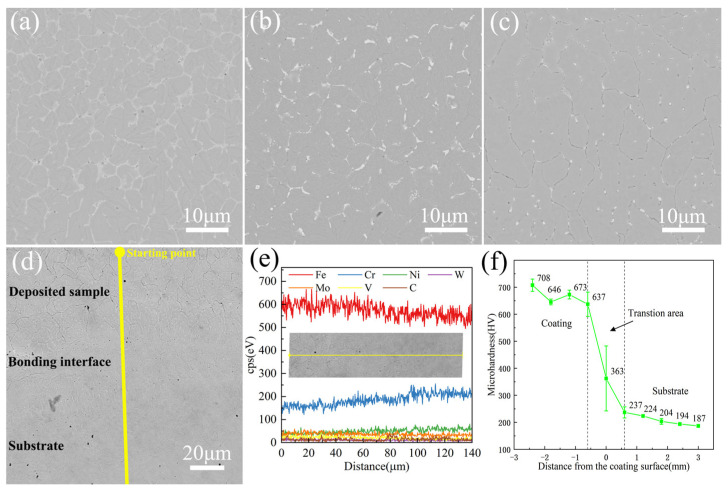
Sample M-1: microstructure of (**a**) top, (**b**) middle, (**c**) bottom, and (**d**) bonding zone, (**e**) line scanning EDS results of bonding zone and (**f**) microhardness distribution curve of M-1.

The evolution of microstructure is affected by the mass transfer, heat transfer, phase transformation, melt flow, and latent heat of crystallization during the rapid solidification process [42]. Figure 8 shows the microstructure of different regions in the deposition direction of the representative sample M-1: the top, middle, bottom, and binding zone of the deposition layer. The top and middle of the sample are mainly equiaxed crystals. A large number of acicular martensite pieces are evenly distributed inside the grains, and discontinuous net white carbides are distributed between the grains, which is consistent with the analysis results of XRD. A comparison reveals that the grains at the top are significantly finer than those in the middle, mainly due to the direct contact of the top deposition layer with air, resulting in a large degree of supercooling and rapid solidification, which limits further grain growth. The size, morphology, and distribution of carbides are also affected by the solidification rate [43]. Since the bottom of the deposited layer is connected to the matrix, the temperature gradient is large, and the solidification rate is slow, leading to the smaller tendency for component supercooling. The carbides at the bottom are different from the network at the top and middle, but are randomly distributed at the grain boundary and within the grains in the form of particles, with a size of about 1 μm. The granular carbides have a pinning effect on the grain boundary and can hinder the further growth of the grains. The EDS line scanning results from the bottom of the deposition layer to the thermal response zone of the substrate are shown in Figure 8e. A small amount of elemental migration was found at the interface, which is manifested in the decrease in Fe and V content and the increase in Gr and Ni content, consistent with the composition difference between M2 and 316 L stainless steel. Figure 8f shows the hardness distribution of the M2 deposition layer along the deposition direction. The average hardness of the deposition layer increases from 187 HV to 708 HV. The higher hardness in the bonding zone is attributed to the formation of martensite and the dispersion of granular carbides. SEM analysis shows that the grain refinement at the top of the deposition layer increases the volume fraction of grain boundaries, hindering dislocation motion better. At the same time, the amount of carbide precipitation at the top of the coating increases significantly, resulting in a further increase in hardness.

**Figure 9:**
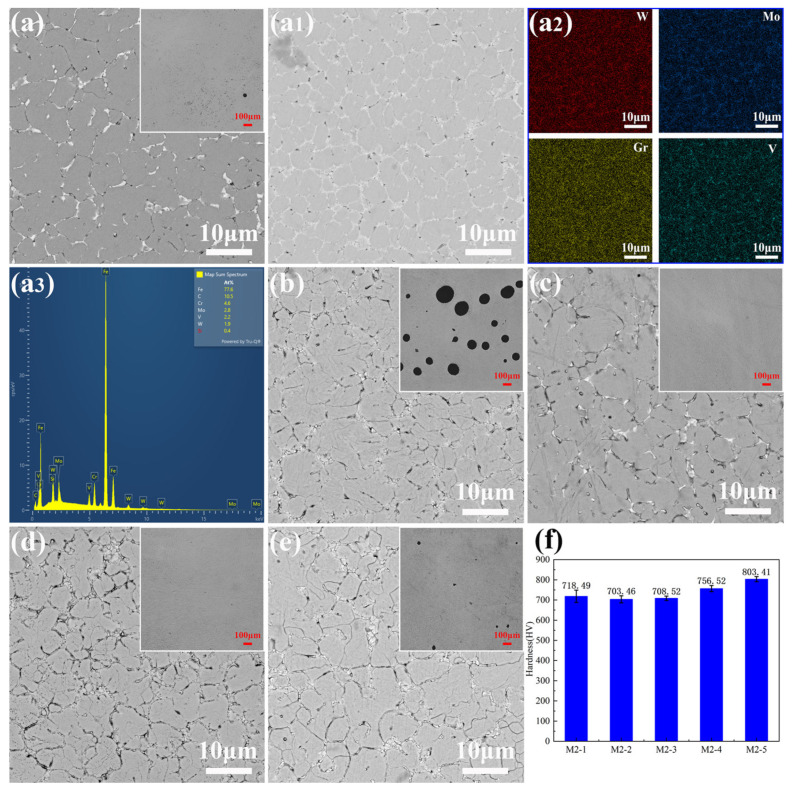
Microstructure and microhardness: (**a**,**a1**) M-1, (**a2**) element mapping of M-1, (**a3**) element composition of M-1, (**b**) M-2, (**c**) M-3, (**d**) M-4, (**e**) M-5, and (**f**) microhardness of deposited samples.

The microstructure and hardness of the top of the M2 HSS samples deposited with different scanning strategies and overlap rates perpendicular to the deposition direction are shown in Figure 9, with the low-magnification optical image of the corresponding samples in the upper right corner. The microstructures of the top sections of the five sample groups exhibit similarities, all presenting as equiaxed grains. This indicates that neither scanning strategies nor overlap rates significantly affect the basic microstructure composition of the samples. The temperature gradient at the top of the sample is small, leading to rapid solidification. At the same time, the solute atoms are enriched, which results in an increasing tendency towards composition undercooling. The sufficiently large region of composition undercooling allows the crystal to grow in the form of dendrites. However, due to the rapid solidification rate in DED, a large number of crystals do not have sufficient time to grow further, maintaining their original equiaxed morphology. The image does not show significant grain orientation and texture differences resulting from varying scanning strategies. This may be due to the rapid melting and solidification cycles during the deposition process, altering the local heat flow direction, which causes the grain orientation of the sample to be rearranged, resulting in no uniform orientation of the sample grains [28]. According to the grain boundary area ratio of the samples in Table 6 to estimate the grain size, there is no significant difference in the grain size of the samples with different scanning strategies. However, the grain size of sample M-4 is notably smaller than that of M-3 and M-5, indicating that a higher overlap rate leads to a higher energy density absorbed by the melt pool, which slows down the cooling rate and provides more time for grain growth and coarsening. Acicular martensite is observed on the surface of all samples, contributing to the improved strength and wear resistance due to the high hardness and strength of martensite. Moreover, there are many white short rod-shaped and dendritic carbides at the grain boundary, while fine granular carbides are distributed within both the grains and at the grain boundary, consistent with XRD results. The carbides in M-4 and M-5 are observed to form clusters, and the carbides of M-3 are more uniformly distributed, which might influence the wear resistance of the samples. In order to further analyze the microstructure of the sample M-1, EDS analysis (Figure 9(a2)) was carried out so that elements such as Mo, W, V, and Gr become concentrated in the same areas, indicating their segregation to the equiaxed grain boundaries and binding with carbon to form a discontinuous network of carbides. Additionally, regions with higher V contents are observed to exhibit a dispersed granular distribution, suggesting that more V is concentrated in individual granular carbides, and MC-type carbides are rich in V [44], leading to the inference that these are hard MC-type carbides. The primary elements detected in the deposited samples include Fe, Mo, V, and Gr, with a notably higher carbon content, which is attributed to carbon’s lightweight nature and its susceptibility to contamination from the environment, making it difficult to perform precise quantitative analysis of carbon using EDS.

No cracks were found in the five groups of deposited samples, but there were pores. The pores were prone to stress concentration when subjected to force, causing crack propagation. Table 6 presents the porosity rates of each sample group, and it is anticipated that the compressive properties and wear resistance will be affected by pores. The formation of pores and voids in additive manufacturing can be attributed to three mechanisms. First, the excessive input energy density induces the melting of the keyhole mode. The steam cavity formed by metal evaporation can easily collapse, or steam retention can form spherical holes. Secondly, gas trapped within the gas-atomized powder particles, which remain in the sample as small spherical pores post-melting, and protective gas being trapped within the melt pool, form gas pores. Third, insufficient fusion between the melt pool and the previously deposited layer can cause irregular voids [45]. There are obvious pores on the surface of M-2 and M-5, but the main reasons for the formation of pores are different. M-2 with a longer unidirectional deposition path undergoes more pronounced cooling due to the longer melting and cooling cycles, which inhibits the release of gasses, while allowing residual tensile stress to accumulate, causing the pores to expand. Moreover, when the deposition is parallel to the axial direction, the molten pool is gradually pressed to the circumferential direction under the influence of gravity, preventing the timely filling of internal pores. In contrast, the high overlap rate of M-5 results in a greater laser energy density, leading to the melting of the keyhole mode. The selective evaporation of the alloy melt forms vapor cavities that collapse into voids, while retained vapor forms numerous spherical pores. Furthermore, the deposited samples with a cross-scanning strategy and a lap rate of 40% and 50% have higher density. The specific performance of the number of pores is worse and the size is less than 8 μm. It is speculated that the residual gas within the powder particles is the primary cause of the pores.

Hardness is primarily influenced by the average grain size, the content of strengthening phases, and the relative density [46]. Corresponding to the microstructure analysis, the hardness histogram of the deposited sample indicates that the hardness difference in the samples with different scanning strategies is less than 2.1%. The grain size of the sample is insensitive to the scanning strategy. Consequently, the average hardness values of the first three groups of samples are relatively similar, and the uneven distribution of carbides in M-1 may explain the more dispersed hardness values. With the increase in overlap rate, the hardness decreases first and then increases. The microstructure size of M-3 increases significantly with the increase in overlap rate, which further reduces the effect of fine grain strengthening and leads to a decrease in microhardness. Although the M-5 sample has a larger grain size and contains obvious pores, its microhardness is higher, which is attributed to the significant increase in the number of thermal cycles, promoting the decomposition of residual austenite, the formation of tempered martensite, and the precipitation of a large number of finely dispersed secondary carbides [47].

### 3.4. Compressive Property

**Figure 10:**
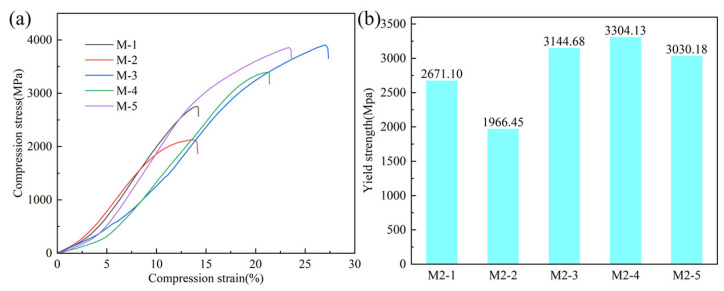
Compressive properties of the samples: (**a**) stress–strain curve and (**b**) yield strength.

The stress–strain curve and yield strength of the unidirectional mechanical compression test are shown in Figure 10a. The deposited samples with cross-layer scanning strategies exhibit significantly better compressive strength than those with axial and circumferential scanning. Previous studies have shown that the interlayer rotation of the deposition path significantly affects the microstructure and mechanical properties of deposited samples [48], and this round of testing further confirms that conclusion. Based on microstructural analysis, although the grain size of the cross-scanned samples is larger than that of the axially and circumferentially scanned samples, the metallurgical bonding between the layers is closer in the cross-scanned samples, while the axially and circumferentially scanned samples have more defects and lower density, especially in M-2, which contains a relatively high number of pore defects that weaken its compressive properties. With the increase in overlap ratio, the compressive strength of the samples deposited by the same scanning strategy increases first and then decreases slightly. Firstly, the increase in overlap rate extends the scanning time per layer, increasing the number of thermal cycles, which promotes the precipitation of higher amounts of tempered martensite and secondary carbides, enhancing the compressive strength of the sample. However, the overlap rate is further increased, resulting in high input laser energy density and serious metal selective evaporation. Finally, the number of pores in M-5 increases, and the continuity of load transfer is destroyed under compression, which enhances the local deformation and uneven distribution of stress. Overall, the high-speed steel deposited by the cross-scanning strategy and 50% overlap rate has the best compressive strength, and its compressive strength is 3904 MPa. Figure 10b shows that the yield strength of M-2 is the lowest due to the presence of significant porosities. With the increase in overlap ratio, the yield strength of the samples gradually decreases, possibly due to the formation of tensile residual stress in the remelted zones, which improves the ability of the sample to resist micro-deformation [49]. The grain size of the M-4 sample is the smallest, and the grain boundary strengthening effect is the most significant, showing the highest yield strength. In summary, the cross-scanned M-3 samples exhibit both high yield strength and the best compressive strength.

### 3.5. Wear Resistance

**Figure 11:**
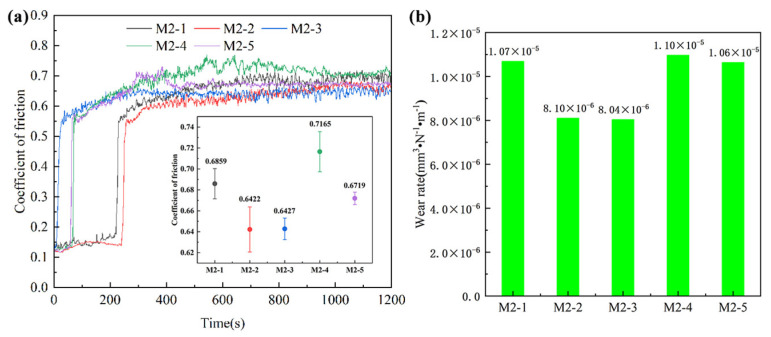
Wear resistance of the samples: (**a**) friction coefficient curve and (**b**) wear rate.

The friction coefficient curve and volume wear rate obtained from wear tests are shown in Figure 11. The friction coefficient is proportional to the actual contact area between the two friction pairs. Due to the microscopic roughness of the two friction surfaces, the actual contact area is small at the initial stage. The other four groups of samples except for M-3 showed a running-in process with a low friction coefficient at the initial stage. As the contact area between the Al_2_O_3_ ball and the sample changes rapidly from point contact to surface contact, the friction coefficient increases sharply. In the later stages of stable friction coefficient variation, the friction coefficient curves of the five samples exhibit an overall upward trend. The surface of the friction pair is continuously crushed under the continuous action of fixed load, which increases the real contact area between the two friction pairs, thereby increasing the friction coefficient. Additionally, there may be an influence of friction heat. Friction heat contributes to the plastic deformation of the sample, while the detachment of wear debris under stress worsens wear resistance, resulting in a higher friction coefficient. Zurcher et al. [50] found that the scanning strategy had no significant effect on wear resistance, but this test’s results showed that although M-2 had obvious holes, it had a lower friction coefficient and better wear resistance than M-1. Different from people’s general understanding, pores are beneficial to the wear resistance of samples under certain conditions, which is attributed to the fact that pores can collect wear debris and reduce further wear. At the same time, areas near porosities are more prone to plastic deformation, reducing contact pressure [51]. The volume wear rate shows the same trend as the friction coefficient, where the wear resistance increases first and then decreases with the increase in overlap rate. Combined with the previous hardness test results, it is found that there is no direct correlation between wear resistance and hardness, and it is speculated that it is related to the type, size, content, and distribution of phases. Consequently, the optimal wear resistance of M-3 can be attributed to the uniform distribution and appropriate size of the carbides. As the input energy density continues to increase, the carbides grow excessively, and the retained austenite content decreases, resulting in M-5 becoming hard but brittle. Large-sized carbides are more likely to peel off during continuous wear, causing more severe scratch damage to the sample surface and ultimately reducing wear resistance [52]. In conclusion, the samples deposited by scanning strategies 2 and 3 have the best wear resistance at a 50% overlap rate, and the volume wear rate is about 27% lower than that of M-4. However, M-2 has obvious hole defects, so M-3 is the sample with the densest structure and the best wear resistance.

**Figure 12:**
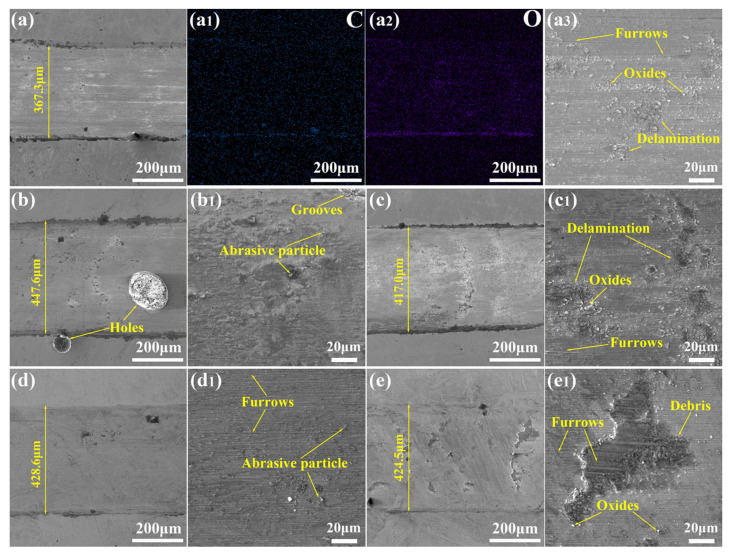
Wear surface morphologies: (**a**,**a3**) M-1, (**a1**,**a2**) element mapping on the worn surface of M-1, (**b**,**b1**) M-2, (**c**,**c1**) M-3, (**d**,**d1**) M-4, and (**e**,**e1**) M-5.

The hardness of the friction pair is significantly higher than that of the sample, resulting in plastic deformation on the friction surface as the mill ball presses into the softer sample during the wear test, and oxidation reactions may occur. Figure 12 shows the wear scar surface morphologies of the five sample groups, where the significant differences in wear patterns suggest different wear mechanisms. Notably, M-1 exhibited the narrowest wear scar (approximately 367 μm), but its wear depth was the greatest (about 5 μm), leading to a relatively high wear rate. When the grinding ball indents the sample, the normal load exerted on the surface causes a plowing effect. Due to the relatively low hardness of M-1, deep grooves were observed on its wear surface. Additionally, there was a small amount of wear debris particles on the surface of M-1. During the wear process, the carbide gradually becomes granular wear debris after continuous grinding and extrusion. Combined with element EDS analysis, the content of oxygen in the wear area was significantly increased compared with that in the non-wear area, indicating that oxidative wear occurred. Moreover, the content of carbon and oxygen in the accumulation of wear debris was higher, indicating that the oxide wear debris and the wear-resistant carbides were squeezed to sides by the mill ball. Adhesive wear caused by plastic deformation occurred obviously in M-1 and M-3, with M-3 exhibiting more pronounced layering. In addition, M-3 contained a small amount of wear debris and shallow grooves, indicating that the wear mechanism of these two samples includes oxidation wear, adhesive wear, and abrasive wear, but the wear rate is significantly different. M-1 experienced primarily abrasive wear, while M-3 is dominated by adhesive wear. The wear surface of M-2 exhibited poor flatness, containing pores and pits, while carbide particles embedded in the wear matrix slow the progression of wear resulting in a lower wear rate. The first three groups of samples have obvious wear debris accumulation on both sides of the wear scar, while the higher hardness of M-4 and M-5 led to less pronounced plastic deformation and minimal wear debris accumulation. Their surfaces displayed typical furrow damage patterns. At the same time, M-5 has a serious collapse due to the lower toughness. Therefore, the dominant wear mechanism for M-4 and M-5 was abrasive wear.

## 4. Conclusions

In this work, M2 HSS powder was deposited on a 316L curved surface by DED. The effects of scanning strategy and overlap ratio on the microstructure, hardness, compressive property, and wear resistance of the samples were studied. The following conclusions were reached:

(1) The M2 powder produced by gas atomization exhibits good sphericity and uniform elemental distribution. As the deposition height of the samples increases, the grain size gradually decreases, and the carbides undergo a transition from granular to short rod and discontinuous network forms, with hardness increasing from 187 HV in the matrix to 708 HV. With the increase in overlap ratio, the grain size initially increases and then decreases, and the hardness first decreases and then increases.

(2) The cross-scanning deposition strategy significantly improves the compressive performance by reducing the porosity defects of the samples. Additionally, the compressive strength of the samples initially increases with the overlap ratio before experiencing a slight decrease. The M-3 sample demonstrates high yield strength and optimal compressive strength, with a compressive strength of 3904 MPa.

(3) The wear resistance is not directly related to the scanning strategy, but is related to the content and size of the phase. The wear rate decreases and then increases as the overlap ratio increases. The M-3 sample shows the dense microstructure and the best wear resistance, its wear rate is just 8 × 10^−6^ mm^3^·N^−1^·m^−1^, and the wear mechanisms primarily include adhesive wear, slight oxidative wear, and abrasive wear.

(4) By optimizing processing parameters, M2 high-speed steel with no significant defects, excellent compressive strength, and superior wear resistance was successfully fabricated. This study provides a reference for the control of cladding layer quality and performance optimization through the adjustment of processing parameters. Moreover, it highlights that utilizing DED technology for the production of roller die cutters can not only shorten production cycles and reduce costs, but also facilitate the fabrication of wear-resistant, long-lasting roller die cutters.

## Figures and Tables

**Table 1:** Chemical composition (in wt.%) of M2 HSS powder.

Elements	Cr	Mn	Mo	Si	V	W	C	S	P	Fe
Standard content (%)	3.8–4.4	0.15–0.4	4.5–5.5	0.2–0.45	0.2–0.45	5.5–6.75	0.8–0.9	≤0.03	≤0.03	Bal.
Content (%)	3.99	0.27	4.56	0.43	1.76	5.68	0.819	0.007	0.013	Bal.

**Table 2:** Chemical composition (in wt.%) of 316 L steel bar.

Elements	Cr	Mn	Mo	Si	C	Ni	S	P	Fe
Standard content (%)	16–18	≤2	2–3	≤1	≤0.08	10–14	≤0.03	≤0.045	Bal.
Content (%)	16.32	1.01	2.01	0.35	0.01	10.06	0.08	0.034	Bal.

**Table 3:** Processing parameters of deposited samples.

No.	Scanning Strategy	Overlap Ratio
M-1	1#	50%
M-2	2#	50%
M-3	3#	50%
M-4	3#	40%
M-5	3#	60%

**Table 4:** Wear resistance test parameters.

Medium Type	Medium Diameter (mm)	Load (N)	Frequency (Hz)	Time (min)	Distance (mm)
Al_2_O_3_	12	5	10	20	10

**Table 5:** Cumulative distribution of three wet particle size tests.

Q3(x) (%)	(a)	(b)	(c)
D10 (μm)	85.21	85.54	84.24
D50 (μm)	130.49	130.81	129.94
D90 (μm)	176.63	176.96	176.63

**Table 6:** Proportion of grain boundary area and the amount and area ratio of pores.

No.	Proportion of Grain Boundary Area (%)	Amount of Pores	Area Ratio of Pores (%)
M-1	8.38	6	0.34
M-2	9.85	25	33.49
M-3	7.58	2	0.01
M-4	12.95	4	0.03
M-5	10.3	6	1.09

## Data Availability

The data presented in this study are available on request from the authors due to some of the data involves privacy.
